# Are Patients Aged 18–25 Reviewed After One Week of Starting a Selective Serotonin Reuptake Inhibitor in a Primary Care Setting?

**DOI:** 10.1192/bjo.2025.10572

**Published:** 2025-06-20

**Authors:** Jesmine Dhooper, Lisa Boruch

**Affiliations:** 1 Dartford and Gravesham NHS Trust, London, United Kingdom; 2 Nottinghamshire Healthcare NHS Foundation Trust, Nottingham, United Kingdom

## Abstract

**Aims:** The National Institute for Health and Care Excellence (NICE) recommends that individuals aged 18–25 or those at increased risk of suicide should be reviewed within one week of initiating or increasing a selective serotonin reuptake inhibitor (SSRI) dose. This study aimed to assess compliance with these guidelines in primary care, identify barriers to timely reviews, and evaluate changes following a previous audit.

**Methods:** A retrospective review was conducted using SystmOne to identify patients aged 18–25 who started an SSRI between 1 December 2023 and 15 July 2024 in a Nottingham GP surgery. Data collected included the time from SSRI initiation to a booked and completed review, as well as instances of non-attendance (DNA). Findings were compared with a prior audit (1 August–24 November 2023) to assess improvements and ongoing challenges. Following the first audit cycle, results were shared and discussed within the practice, prompting greater awareness from all members of the multidisciplinary team (MDT) upon current guidance and performance.

**Results:** In the initial audit, none of the 21 eligible patients had a review booked within one week, with an average booking time of 20 days and 30 days to an actual review. In the re-audit, 36 eligible patients were identified, with a slight improvement in booking time (19 days) and review completion (23 days). Three patients (8.3%) had a review scheduled within the recommended one-week timeframe.

The main barrier remained appointment availability, with a shortage of GP slots limiting one-week follow-ups. High DNA rates persisted, with 14 patients missing their reviews in the re-audit. No standardised approach to DNAs was implemented, with some patients receiving multiple recall attempts and medication re-issues, while others had no further action documented.

**Conclusion:** Over this one-year period, noticeable improvements were observed in both booked and actual SSRI review times. However, most patients still did not receive a timely review. Limited appointment availability and inconsistent follow-up for DNAs remained significant challenges. Expanding the role of other healthcare professionals, such as pharmacists, to conduct initial medication reviews could improve guideline compliance and reduce GP workload. Establishing a standardised protocol for DNAs, ensuring a set emergency medication supply and a timely follow-up, is essential to improving patient safety and treatment outcomes.

